# Innovative, complementary and alternative therapy in inflammatory bowel diseases: A broad 2020s update

**DOI:** 10.3389/fgstr.2022.1022530

**Published:** 2022-10-17

**Authors:** Letizia Masi, Cristina Ciuffini, Valentina Petito, Laura Francesca Pisani, Loris Riccardo Lopetuso, Cristina Graziani, Daniela Pugliese, Lucrezia Laterza, Pierluigi Puca, Federica Di Vincenzo, Marco Pizzoferrato, Daniele Napolitano, Laura Turchini, Valeria Amatucci, Elisa Schiavoni, Giuseppe Privitera, Laura Maria Minordi, Maria Chiara Mentella, Alfredo Papa, Alessandro Armuzzi, Antonio Gasbarrini, Franco Scaldaferri

**Affiliations:** ^1^ Centro per le Malattie dell’Apparato Digerente (CEMAD), Dipartimento di Scienze Mediche e Chirurgiche, Fondazione Policlinico Universitario “A. Gemelli” IRCCS, Roma, Italy; ^2^ Dipartimento Universitario di Medicina e Chirurgia Traslazionale, Università Cattolica del Sacro Cuore, Roma, Italy; ^3^ Dipartimento di Biotecnologie e Bioscenze, Università degli Studi di Milano-Bicocca, Milano, Italy; ^4^ Dipartimento di Medicina e Scienze dell’Invecchiamento, Università degli studi di “G. D’Annunzio” Chieti-Pescara, Chieti, Italy; ^5^ Center for Advanced Studies and Technology (CAST), Università degli studi di “G. D’Annunzio” Chieti-Pescara, Chieti, Italy; ^6^ Dipartimento di Diagnostica per Immagini, Radioterapia Oncologica ed Ematologia, Fondazione Policlinico Universitario “A. Gemelli” IRCCS, Roma, Italy; ^7^ Nutrizione Clinica, Dipartimento di Scienze Mediche e Chirurgiche, Fondazione Policlinico Universitario “A. Gemelli” IRCCS, Roma, Italy; ^8^ IBD Center, IRCCS Humanitas Research Hospital, Rozzano, Italy

**Keywords:** crohn’s disease, ulcerative colitis, innovative therapies, complementary and alternative medicine, small molecules, biologic drugs

## Abstract

Inflammatory bowel diseases (IBD) are chronic disabling conditions with a complex and multifactorial etiology, which is still not completely understood. In the last 20 years, anti-TNF-α antagonists have revolutionized the treatment of IBD, but many patients still do not respond or experience adverse events. Therefore, new biological therapies and small molecules, targeting several different pathways of gut inflammation, have been developed of which some have already been introduced in clinical practice while many others are currently investigated. Moreover, therapeutic procedures such as leukocytapheresis, fecal microbiota transplant and stem cell transplantation are currently being investigated for treating IBD. Lastly, complementary and alternative medicine has become a field of interest for gastroenterologist to reduce symptom burden in IBD patients. In this comprehensive and updated review, a novel classification of current and developing drugs is provided.

## Introduction

Inflammatory bowel diseases (IBD), mainly Crohn’s disease (CD) and ulcerative colitis (UC), are lifelong conditions with a relapsing and remitting course that results from dysregulated immune responses against gut microbiota antigens. Despite many treatments for IBD, a number of patients fail to respond to long-term treatment, which has a negative impact on their quality of life. The etiology of IBD is still not completely understood and multifactorial, involving many susceptibility genes and environmental factors (1, 2).

In the last two decades, the introduction in clinical practice of monoclonal antibodies directed against pro-inflammatory cytokine tumour necrosis factor alpha (TNFα), e.g., infliximab, adalimumab, golimumab, certolizumab pegol, revolutionized IBD management, because anti-TNFα drugs induce and maintain clinical remission and mucosal healing (3), which reduce the need for corticosteroids, hospitalization and surgery; this improved health related quality of life (HRQOL) of IBD patients (4–13). Unfortunately, anti-TNFα therapy has been associated with infectious complications (14), and clinical efficacy is limited by a high rate of primary and secondary non-responsiveness (30%-40%). Loss of response could be a consequence of antibody production against the TNFα antagonists that can neutralize the drug, accelerate its clearance or induce infusion reactions (15, 16). For these reasons, new therapies with a better safety profile targeting different working mechanisms of inflammation, e.g., vedolizumab and ustekinumab, were approved for clinical practice and many others are presently studied (17, 18).

Small-molecule drugs (SMDs) are investigated to treat IBD, because of the high rate of non-response or loss of response to marketed drugs, and due to their invasive route of administration. In particular, SMDs have a low molecular weight (< 1 kDa) enabling them to diffuse through cell membranes to reach their intracellular targets (19). These drugs carry several advantages including lack of immunogenicity, short half-lives and oral administration (20). For every mechanism, SMDs are discussed in the second part of each paragraph.

Following our increasing knowledge about IBD pathogenesis, and specifically the role of an abnormal immune response against gut microbiota antigens, procedures like leukocytapheresis, fecal microbiota transplant and stem cell transplantation, which already proved to be effective, are also proposed as a therapeutic strategy in IBD (21–23).

Moreover, the use of complementary and alternative medicine (CAM) by gastroenterologists has become a common adjuvant to conventional medicine in the management of IBD patients (24–26).

This review aims to provide a broad overview of complementary and alternative therapies in IBD and to summarize the scientific evidence of currently used and developing drugs, providing a new comprehensive classification.

## Methods

### Search strategy

The literature search was conducted in PubMed (https://pubmed.ncbi.nlm.nih.gov) and Scopus (https://www.scopus.com) databases without restriction on publication period, using the following search string: (“IBD”, “ulcerative colitis”, Crohn’s disease) AND (“innovative therapy”, “complementary therapy”, “alternative therapy”). The “AND” operator was used to create all possible combinations of selected terms. ClinicalTrials.gov (https://clinicaltrials.gov) contains information about medical studies in human volunteer and was used to search for ongoing clinical trials.

### Study selection

The initial screening of documents, using abstract and titles, was carried out including only English-language research articles, while articles without full text and abstract, duplicates studies, conferences, review articles, and editorial reports were excluded.

The selected clinical trials included in this review considered new small molecules or antibodies still in the experimental phase II, II or III used in IBD (UC and CD) patients.

In addition, the efficacy and safety of complementary and alternative therapies and how these could be a supplement in the treatment of IBD were evaluated.

Following the initial internet search, a total of 96 studies were retrieved by databases and following elimination of duplicates 88 articles remained. Out of the remaining studies, 11 records were excluded after review of their titles and abstracts and because not available in English language. At the end, 80 studies were included in this review, as 3 articles from other sources have been added.

**Figure 1:**
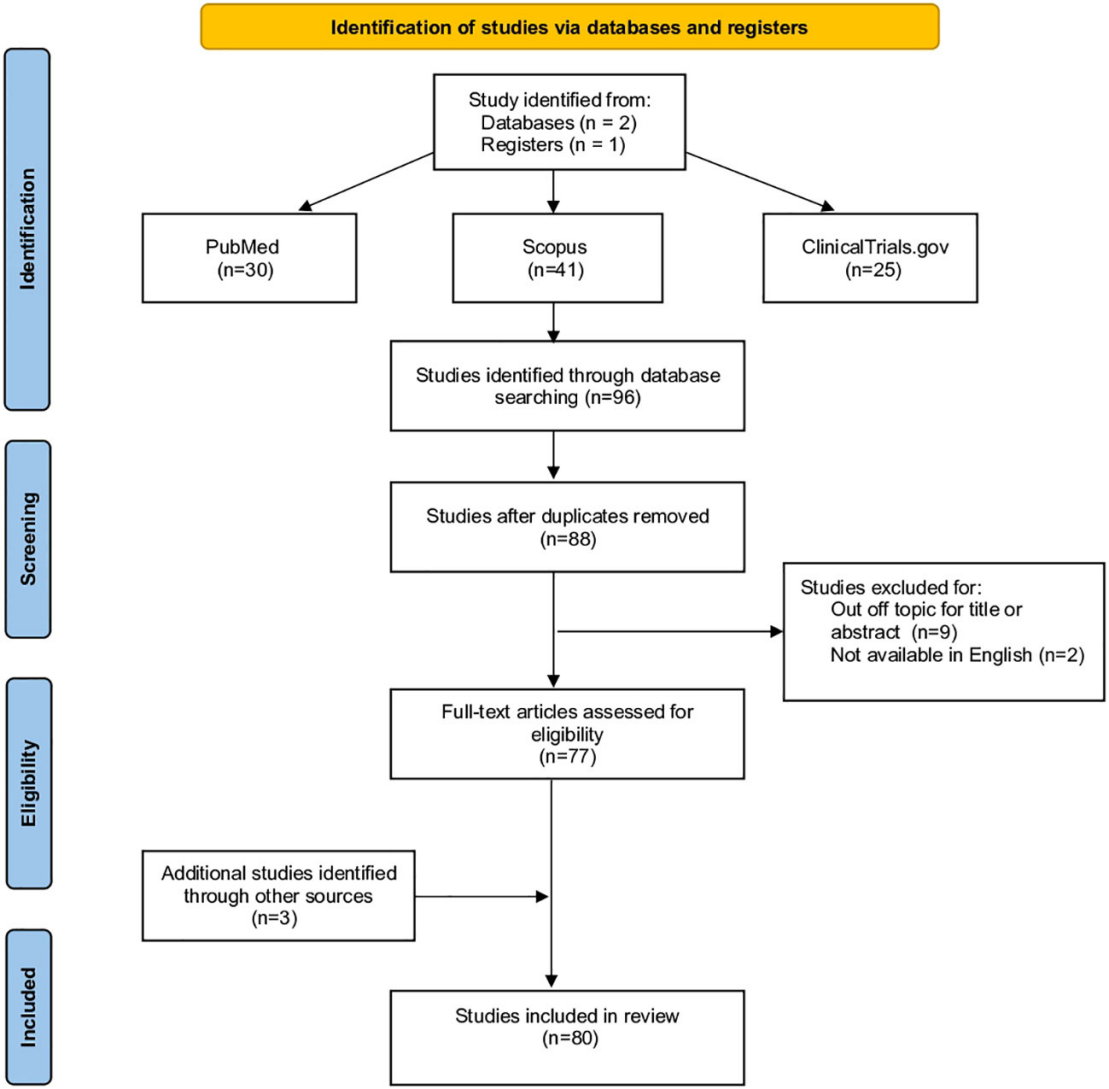
PRISMA 2020 flow diagram for new systematic reviews which included searches of databases and registers only.

The Preferred Reporting Items for Systematic Review and Meta-Analysis (PRISMA) was used as a guideline for including and reporting the studies and meta-analysis that we have included in this review (**Figure 1**).

## Current drug targets and innovative therapies

### Pro-inflammatory cytokine neutralization

IL-23 and IL-12 are proinflammatory cytokines that promote differentiation of naive CD4+ precursor T cells into T-helper 1 and T-helper 17; their inhibition has become a novel therapeutic target in IBD (27). Ustekinumab, a fully human immunoglobulin G1 kappa monoclonal antibody against the p40 subunit of IL-12 and IL-23 (28), has been approved for both induction and maintenance of clinical response and the remission in moderate-to-severe CD in patients with anti-TNF-α therapy failure (18). In 2019, a phase III study demonstrated that ustekinumab reached the same clinical response in UC that led to the approval to treat moderate to severe UC (29).

A new class of antibodies, including risankizumab, brazikumab, mirikizumab and guselkumab, that target solely IL-23 by linking its p19 subunit and that appears to have a potentially greater anti-inflammatory activity associated with an even safer profile, are currently investigated in phase II/III trials and showed promising results as induction and maintaining therapy in both CD and UC (30–34). One of them, Risankizumab, has been approved for the treatment of moderate to severe Crohn’s Disease (35–38). Guselkumab instead has been approved for the treatment of psoriasis and psoriatic arthritis (39).

PTG-200 is a new SMDs that antagonizes the IL-23 receptor (IL-23R). A phase I study has shown its efficacy in ameliorating colitis in rat models and a phase II trial for CD is currently recruiting patients (40).

Together with IL-12 and IL-23 overexpression, higher levels of IL-18 and IL-36 family members were described within the inflamed mucosa of IBD patients (41, 42). The IL-18 antibody GSK1070806 passed safety evaluation in a phase I clinical trial and is currently being investigated in phase I/II clinical trial to treat CD (43). Spesolimab, a humanized monoclonal IgG1 antibody, directed against the IL-36R, has shown clinical activity in patients with moderate-to-severe active UC, who failed previous biologic treatments (44). The long-term efficacy and safety of the IL-36R blockade with spesolimab are studied in a subsequent open-label phase II trial with UC patients who completed previous trials (45). Furthermore, spesolimab is undergoing phase II evaluation in patients with fistulising CD (46, 47).

### Anti-inflammatory cytokines/pathway stimulation

#### Interleukin 10 and interleukin 22 agonists

Interleukin 10 (IL-10) is a cytokine with anti-inflammatory effects that inhibits both the antigen presentation and the subsequent release of pro-inflammatory cytokines and IL-22 attenuates intestinal inflammation, inducing the production of a mucine membrane by goblet cells (48). AMT-101 is a novel, GI-selective, investigational oral recombinant biologic fusion protein of human interleukin 10 (hIL-10), which is under development to treat IBD. AMT-101 will be evaluated in two phase II clinical trials covering a wide spectrum of UC patients: a monotherapy study to evaluate the efficacy and safety of the drug compared with a placebo (49) and a randomized, double-blind study in UC patients with an ileal pouch-anal anastomosis (IPAA) post-colectomy who have developed pouchitis (50). UTTR1147A is a recombinant fusion protein, targeting intestinal epithelial IL-22 receptors to promote mucosal repair. Promising results from a phase I clinical study showed improved clinical and pharmacodynamic responses in patients with UC treated with UTTR1147A compared with placebo (51). Currently, a phase II study is investigating the long-term safety and tolerability of UTTR1147A in participants with moderate to severe UC or CD (52).

#### STAT3 pathway stimulation

Over the past decades, scientists have investigated the role of microRNAs as key players in regulating immune responses.

microRNA showed the stimulation of anti-inflammatory pathways; in particular, miR-124, which can dampen inflammation, was previously found to be reduced in patients with certain autoimmune diseases. Importantly, increasing levels of miR-124 were found to inhibit intestinal inflammation by suppressing the activity of a main regulator of the inflammatory response, called STAT3 (53). ABX464, a small molecule, originally developed as an inhibitor of HIV replication, was shown to promote the production of miR-124 in immune cells. This drug has demonstrated safety and profound anti-inflammatory activity in preclinical trials. More specifically, in a phase IIa clinical trial, ABX464-101 showed effectiveness to treat UC (54). Based on the good safety profile and promising efficacy results obtained for ABX464 in UC, the initiation of a pivotal phase II trial for the treatment of subjects with moderate to severe active CD has been encouraged (55).

### Blocking leukocytes trafficking

#### Anti-integrins

Another mechanism of IBD pathogenesis is leukocyte migration to gut mucosa mediated by the interaction between integrins on leukocyte surfaces and their ligands on endothelial and epithelial cells (56). Natalizumab, a humanized IgG4 anti-α4-integrin monoclonal antibody that inhibits both α4β7-integrin/mucosal addressin-cell adhesion molecule-1 (MadCAM-1) interaction and α4β1/vascular-cell adhesion molecule-1 (VCAM-1) binding, was the first drug targeting leukocyte trafficking in IBD. Despite natalizumab inducing clinical response and remission in CD, it is associated with an increased risk of progressive multifocal leukoencephalopathy (PML) due to its non-selective nature (57, 58). Vedolizumab, a fully humanized IgG1 monoclonal antibody binding α4β7 integrin, which ligand MadCAM-1expression is mainly restricted to the intestine, has shown to be effective and safe in moderate to severe UC and CD, especially in anti-TNFα naive patients and its good safety profile led to its approval in clinical practice in 2014 (59–61). Several new antibodies directed against integrins are currently being studied. Abrilumab, an anti-α4β7 antibody, induced clinical remission in UC, but did not reach statistical significance in CD in phase II trials (62, 63). Etrolizumab targets the β7 subunit of integrins α4β7 and αEβ7 that bind respectively MadCAM-1 and E-cadherin. In phase III trials, it has been more effective than placebo inducing clinical and endoscopic improvements in IBD (64). PN-943, an oral α4β7 integrin antagonist, is currently in a phase II placebo-controlled study to evaluate its safety, tolerability, and clinical efficacy in patients with moderate to severe active UC (65). Ontamalimab, a monoclonal antibody directed against MadCAM-1, a ligand of integrins, has been demonstrated to be effective and safe as induction therapy in UC, but the response and remission rates were not statistically significant in CD (66, 67). Currently, ontamalimab is undergoing phase III evaluation in IBD.

In addition, a new oral small molecule targeting integrins has been developed and investigated. AJM300 is an α4 integrin subunit antagonist that was more effective in a phase II study than placebo to achieve clinical remission and mucosa healing in moderate to severe UC, with no cases of PML (68). A phase III trial on efficacy and safety of an oral dose of AJM300 is active in Japan.

In addition to integrins, other adhesion molecules became therapeutic targets to suppress gut inflammation. Another SMD, with a similar mechanism is alicaforsen, which is an oligonucleotide that binds the mRNA of ICAM-1 (Intercellular Cell Adhesion Molecule-1), another ligand of integrins. Its action results in a reduced translation and expression of ICAM-1 by endothelial cells (69). Alicaforsen presents ICAM-1 mRNA to an RNAse, which enzymatically hydrolyse this mRNA (69). Despite the promising perspective (69–71), results emerging from the trials showed alicaforsen ineffectiveness to induce clinical remission in CD (72, 73). In UC, studies evidenced that alicaforsen enema induced acute and long-term topical improvement in patients with mild to moderate disease with a modest safety profile, but no significant differences were highlighted in clinical outcomes between patients treated with alicaforsen and placebo or mesalazine enemas (74–76). An open-label trial showed positive results in the treatment of pouchitis, and a phase III study on refractory pouchitis has just been completed (77).

#### Sphingosine-1 Phosphate Receptor modulation

Sphingosine-1 Phosphate Receptor modulators (S1PRs) are a class of small molecules that, after the interaction with their ligand, i.e. Sphingosine-1 Phosphate (S1P), cause internalization and degradation of S1P receptors, resulting in lymphocyte sequestration in lymph nodes and blocking their migration to the gut. S1P regulates the migration of naïve and central memory CCR7-positive T cells from the secondary lymphatic organs to the periphery. Fingolimod, a potent non-selective S1PR agonist used in multiple sclerosis, was effective in reducing gut inflammation in rats (78, 79). However, its clinical use in IBD was never been tested for its association with severe adverse events and cases of PML (80, 81). This led to the development of a more selective S1PR with a better safety profile. Ozanimod, an S1P1 and S1P5 modulator, and etrasimod, an oral S1P1, S1P4 and S1P55 modulator, produced positive results in terms of clinical and endoscopic improvement in IBD in phase II and III trials with an acceptable safety profile, since the adverse events reported, as bradycardia or conduction abnormalities, were mostly from mild to moderate. Several phase II and III trials on these selective S1PR agonists in both CD and UC are already ongoing (82–84). Ozanimod received approval for the treatment of moderate to severe UC/CD, because of the results obtained in phase III trials, such as improvement in clinical remission (primary end point) and in all key secondary end point (clinical response, endoscopic gain and mucosal healing) (85). Amiselimod/MT-1303 is a selective S1P1 modulator that has been tested in CD and UC and dose-finding studies have been completed (86, 87).

#### Anti-chemokine

Fractalkine (FKN)/CX3CL1 is a membrane-bound chemokine possessing a chemokine/mucin hybrid structure and a transmembrane domain and has a dual function as an adhesion molecule and a chemoattractant (88). It represents a new type of leukocyte trafficking regulator, which, signaling *via* its receptor CX3CR1 on leukocytes, allows them to migrate directly to effector sites during inflammation (88). E6011, a humanized anti-FKN monoclonal antibody, was evaluated for safety, tolerability, pharmacokinetics, and pharmacodynamics in a phase Ib randomized, double-blind, placebo-controlled trial in patients with moderate to severe CD (89).

### Blocking intracellular pathways

#### Jak Inhibitors

Many cytokines, binding their receptors, induce intracellular phosphorylation and activation of Janus Kinases, a group of receptor-associated tyrosine kinases that convert extracellular stimuli into cellular processes involved in innate and adaptive immunity, through the activation of Signal Transducers and Activators of Transcription (STATs) (90). Given the role of the JAK/STAT pathway also in IBD pathogenesis, a new class of SMDs called JAK inhibitors, is being developed (90). Tofacitinib inhibits preferentially JAK1 and JAK3, which proved to be safe and effective as induction and maintenance therapy in UC, but not in CD in phase II and III clinical trials (91–95). In 2018, tofacitinib was approved in the US for the treatment of moderate to severe UC (90). In a phase II evaluation, filgotinib, a JAK inhibitor especially active against JAK1, and upadacitinib, a JAK1-selective inhibitor, showed good results in terms of clinical and endoscopic remission in CD and UC patients. Filgotinib is now undergoing phase III studies (96); filgotinib and updacitinib have been approved for the treatment of moderate to severe UC (97, 98).

A JAK1 and JAK3 inhibitor called peficitinib showed non-statistically significant efficacy in phase II trials in UC; therefore, further investigation was suspended (90, 99).

SHR0302 is a potent and highly selective JAK1 inhibitor and several late-stage clinical studies are ongoing with both oral and topical dosage forms for several immune-inflammatory diseases including UC and CD (100, 101).

Other drugs belonging to this category, but with different targets, like a JAK3 inhibitor (Pf-06651600) and a TYK2/JAK1 inhibitor (Pf-06700841), are being tested in phase II clinical trials in UC (90).

OST-122 is an oral, gut-restricted and subtype-selective JAK3/TYK2 inhibitor for the local treatment of IBD including UC, CD and, potentially, fibrotic lesions in CD patients. A phase I/II is currently underway to evaluate the safety and tolerability of OST-122 in patients with moderate to severe UC over 28 days. This trial will also explore the OST-122 pharmacokinetics (PK) profile and preliminary therapeutic efficacy through biomarker analysis and clinical, endoscopic and histologic assessments (102).

Deucravacitinib (BMS-986165), an oral selective TYK2 inhibitor is being studied in a wide spectrum of immune-mediated diseases, including psoriasis, psoriatic arthritis, lupus and inflammatory bowel disease (103). A phase II randomized, double-blind, placebo-controlled study is ongoing to establish the safety, efficacy, and biomarker response of BMS-986165 in subjects with moderate to severe UC and CD (104–106).

Unfortunately, JAK inhibitors seem to increase the risk of viral infections, particularly herpes zoster, but an increased risk of malignancy has not been reported (90). TD-1473, is a new pan-JAK inhibitor with the advantage of distribution limited to the gastrointestinal tract, minimizing systemic side effect, as demonstrated in a phase I study on healthy volunteers (107). In another phase I study, TD-1473 also proved clinical and biomarker activity in patients with moderate to severe UC (108). Phase II/III studies are ongoing in IBD.

#### PDE4 and NFκB modulation

Among second messengers, cyclic adenosine monophosphate (cAMP) is fundamental in inhibiting the pro-inflammatory process. Phosphodiesterase 4 (PDE4) is the predominant enzyme that metabolizes and degrades cAMP in inflammatory cells, resulting in the production of pro-inflammatory mediators, such as TNF-α, interferon-γ, and IL-23 (109).

The anti-inflammatory and immunomodulatory potential of PDE4 inhibitors in human leukocytes, endothelium and epithelium are now well documented (110–112).

Apremilast is a PDE4 inhibitor that can induce clinical remission, endoscopic and histological response in UC with an acceptable safety profile (113), although not statistically significant when compared to a placebo.

Laquinimod is another small molecule immunomodulator that, acting on the NF-κB pathway of dendritic cells, causes a reduction of pro-inflammatory cytokine and chemokine production and leucocyte migration. In a phase II trial, laquinimod leads to clinical response and remission in patients with moderate to severe UC (114).

#### Oligonucleotides messenger RNA inhibition

Oligonucleotides represent an emerging category of highly selective targeted agents, which action is mediated by the inhibition/degradation of messenger RNA of different pro-inflammatory cytokines or by bacterial antigen simulation triggering cellular targets for immunomodulation (115). Aside from alicaforsen, already discussed for its anti-trafficking effect, other oligonucleotides that determine the disruption of specific messenger RNA (mRNA) are currently undergoing preliminary investigation. SB012 is a GATA3-specific DNAzyme that can mediate the cleavage of the mRNA of the transcription factor GATA3, responsible for Th2 differentiation and that has proven to be over-expressed in the colonic mucosa of UC patients (116). A phase I/II study on efficacy, pharmacokinetics, tolerability and safety of SB012 enema in patients with active UC is completed but results are still unpublished (115). STNM01 is a double-strand RNA that is directed against the mRNA coding for carbohydrate sulfotransferase 15 (CHST15), an enzyme that mediates the biosynthesis of glycosaminoglycans (GAG), a process involved in the fibrotic evolution of IBD. In a phase I study, a submucosal injection of STNM01 in CD patients with treatment refractory mucosal lesions was safe and able to reduce Simple Endoscopic Score-CD and fibrosis (117). Another drug that deserves a mention is mongersen/GED-0301, an antisense oligonucleotide that mediates the degradation of Smad7 mRNA (118–120). Smad7 is an intracellular protein that inhibits the immunosuppressive cytokine TGF-β and was found to be overexpressed in IBD (121). In phase I and II trials, mongersen had shown safety, tolerability and efficacy in inducing endoscopic response and clinical remission in CD, but a phase III trial was discontinued because the drug was found to be non-effective (115, 118–120).

### Innate immunity modulators

As mentioned above, oligonucleotide-based agents can elicit the innate immunity response linking a member of the Toll-Like Receptor (TLR) family, a group of receptors expressed on the surface of immune cells that are usually activated after the interaction with pathogen-associated molecular patterns (PAMPs) expressed by infectious agents (115). Cobitolimod/DIMS0150 is a single-strand oligonucleotide that mimics bacterial DNA as it is recognized by TLR9, mediating the induction of anti-inflammatory cytokines like IL-10 and IFNα (122). A small study, conducted in 2013, showed that topical administration of cobitolimod determined reduction in colitis activity index and clinical, endoscopic and histologic improvements in UC patients otherwise candidates for colectomy (122). In a randomized, placebo-controlled phase III trial with cobitolimod, a statistically significant improvement in clinical remission with mucosal healing and symptomatic remission was reported, together with a good safety profile at week 4. On the other side, it did not reach statistical significance in inducing remission in UC patients treatment-refractory at week 12 (123). Furthermore, a *post-hoc* analysis showed statistical significance in the rate of clinical remission using patient-reported outcome (PRO) measures (124). Similarly, BL7040 (monarsen) is an orally applied oligonucleotide that modulates the TLR9 that achieved clinical response in a phase II study in patients with moderately active UC (125). FimH, a novel microbiome-derived therapeutic target, is a TLR4 receptor, expressed on *Escherichia coli* and other *Enterobacteriaceae* in a host with CD. The inhibition of FimH adhesion, and consequently intracellular replication of adherent-invasive *E.coli* in epithelial cells, may prevent the establishment of a sub-mucosal infection leading to mucosal inflammation and epithelial barrier disruption (126).

Sibofimloc is a small molecule specifically designed to reduce the inflammatory cascade underlying CD and remain gut-restricted to minimize absorption into the bloodstream. This drug was included in a randomized, double-blind, placebo-controlled, multicenter, phase II study to evaluate its safety and tolerability to prevent the recurrence of intestinal inflammation in up to 96 postoperative participants with CD and the results are under discussion (127).

There is growing evidence that a variety of immune-related diseases, including UC, have an underlying defect or suppression of the innate immune system. QBECO, Site Specific Immunomodulator (SSI) derived from components of inactivated *E.coli*, has been approved in UC and designed to restore innate immune function in the colon and reverse the chronic inflammation and dysbiosis associated with the disease (128). Promising early clinical experience and a translational study with QBECO in UC, showing improved GI barrier function, have led to exploring the safety, efficacy, and tolerability of this novel immunotherapy in subjects with moderate to severe CD (129, 130).

The immune-activating receptor Natural Killer Group 2D (NKG2D) has been implicated in the pathogenesis of IBD through its presence on intestinal cytotoxic lymphocytes and the increased expression of activating ligands on inflamed tissue (131).

In IBD, the expression and function of NKG2D have not been fully characterized, and a recent phase II clinical trial tested the efficacy and safety of tesnatilimab (JNJ-64304500) in patients with active CD but not remission to the standard of care biologic therapy (132, 133).

**Table 1** shows a list of current and emerging targeted therapies for IBD with their main characteristics.

**Table 1:** Current and future therapies in IBD based on mechanisms of action, name of drug and type of molecule (table continues on the following page).

DRUGS CLASSIFIED PER MECHANISMS OF ACTION	NAME OF DRUG	DRUGS CLASSIFIED PER TYPE OF MOLECULE	TARGET		DRUGS CLASSIFIED PER ADMINISTRATION ROUTE	CURRENT STATUS IN CD	CURRENT STATUS IN UC
**PRO-INFLAMMATORY CYTOKINES NEUTRALIZATION**							
	Infliximab		ANTI-TUMOR NECROSIS FACTOR	TNF-α	IV	Approved	Approved
	Adalimumab			TNF-α	SC	Approved	Approved
	Golimumab			TNF-α	SC	—	Approved
	Ustekinumab	ANTIBODIES		IL12/23 p40	IV/SC	Approved	Approved
	Risankizumab		ANTI-INTERLEUKINS	IL23 p19	IV/SC	Approved	Phase 2/3
	Brazikumab			IL23 p19	IV/SC	Phase 2/3	Phase 2
	Mirikizumab			IL23 p19	IV/SC	Phase 3	Phase 2/3
	Guselkumab			IL23 p19	IV/SC	Phase 2/3	Phase 2/3
	GSK1070806	SMALL MOLECULES		IL18	IV	Phase 1/2	—
	Spesolimab			IL36R	IV	Phase 2	Phase 2/3
	PTG-200			IL23R	Oral	Phase 2	—
**ANTI-INFLAMMATORY CYTOKINES/PATHWAY STIMULATION**							
	AMT-101	FUSION PROTEIN	INTERLEUKINS AGONISTS	hIL-10	Oral	—	Phase 2
	UTTR1147A			IL22R	IV	Phase 2	Phase 2
	ABX464	Small molecule	microRNA MODULATION	miR-124	Oral	Phase 2	Phase 2
**BLOCKING LEUKOCYTES TRAFFICKING**							
	Natalizumab			α4 subunit	IV	Approved (limited use)	—
	Vedolizumab			α4β7 integrin	IV	Approved	Approved
	Abrilumab	ANTIBODIES	ANTI-ADHESION	α4β7integrin	SC	Phase 2	Phase 2
	Etrolizumab			β7 subunit	SC	Phase 3	Phase 3
	PN-943	SMALL MOLECULES	S1PR MODULATION	α4β7 integrin	Oral	—	Phase 2
	AJM300			α4 subunit	Oral	—	Phase 3
	Alicaforsen	OLIGONUCLEOTIDE		ICAM-1 mRNA	IV/Enema	Discontinued	Phase 3 (Pouchitis)
	Ozanimod			S1PR1/5	Oral	Approved	Approved
	Etrasimod	SMALL MOLECULES		S1PR 1/4/5	Oral	Phase 2	Phase 3
	Amiselimod	ANTIBODIES	ANTI-ADHESION	S1PR1/5	Oral	Discontinued	Discontinued
	E6011			CX3CL1	IV	Phase 2	—
**INTRACELLULAR PATWHAYS BLOCKADE**							
	Tofacitinib			JAK 1/3	Oral	Phase 2	Approved (US)
	Filgotinib			JAK1	Oral	Phase 2/3	Phase 3
	Upadacitinib			JAK1	Oral	Phase 2/3	Approved
	Peficitinib	SMALL MOLECULES	JAK INHIBITION	JAK 1/3	Oral	—	Discontinued
	SHR0302			JAK1	Oral/topical application	Phase 2	Phase 2
	Pf-06651600			JAK 3	Oral	—	Phase 2
	Pf-06700841			TYK2/JAK1	Oral	—	Phase 2
**INTRACELLULAR PATWHAYS BLOCKADE**							
	OST-122	SMALL MOLECULES	JAK INHIBITION	TYK2/JAK3	Oral	—	Phase 2
	Deucravacitinib			TYK2	Oral	Phase 2	Phase 2
	Apremilast		NFkB MODULATION	PDE4	Oral	—	Phese 2
	Laquinimod			NFκB pathway	Oral	—	Phase 2
	SB012			GATA-3 mRNA	Enema	—	Phase 1/2
	STNM01			CHST15 mRNA	Submucosal injection	Phase 1	—
	Mongersen	OLIGONUCLEOTIDE	mRNA INHIBITION	SMAD7 mRNA	Oral	Discontinued	—
**INNATE** **IMMUNITY MODULATORS**							
	Cobitolimod	OLIGONUCLEOTIDE	TLR9 MODULATION	TLR9	Topical application	—	Phase 3
	BL7040 (Monarsen)			TLR9	Oral	—	Phase 2
	Sibofimloc	SMALL MOLECULES.	MICROBIOME MODULATION	FimH	Oral	Phase 2	—
	QBECO	BIOLOGIC MOLECULES	SITE SPECIFIC IMMUNOMODULATOR	Macrophage	SC	Phase 2	Approved
	Tesnatilimab	ANTIBODIES	NK INHIBITION	NKG2D	SC	Phase 2	—

### Leukocytapheresis

Leukocytapheresis is a non-pharmacologic approach to the treatment of IBD that works by removing activated circulating leukocytes in the colonic mucosa using beads or filters. A selective apheresis filter currently available is the Adacolumn^®^ system (JIMRO, Japan), which contains cellulose diacetate beads that adhere to Fc-gamma and complement receptors on granulocytes and monocytes, selectively removing them without significantly affecting lymphocytes or platelets (21). Adacolumn^®^ significantly suppresses proinflammatory cytokines, reduces neutrophil chemotaxis, causes down-regulation of leukocyte adhesion molecules and reduces neutrophil adhesion to endothelial cells activated by IL-1β; it also causes an adsorptive-dependent increase in anti-inflammatory cytokines such as IL-1Ra, growth factor of hepatocytes and soluble receptors I and II for TNF (134). Randomized phase III trials failed to demonstrate any efficacy of the Adacolumn^®^ for induction therapy in patients with moderate to severe UC or active CD (135, 136). Conversely, two recent trials have shown efficacy in inducing remission for steroid-dependent ulcerative colitis demonstrating a potential role in this subclass of patients (137, 138).

### Fecal microbiota transplant (FMT)

The gut microbiome has a mutual symbiotic relationship with the host, providing, for example, the production of carbohydrates, short-chain fatty acids and vitamins and the maintenance of immune homeostasis. Dysbiosis in IBD, most commonly causes a decrease in commensal bacteria diversity and an increase in bacterial species belonging to *Enterobacteriaceae*; this is generally accepted even though a clear causal link between cause and effect has not yet been established (139). Fecal microbiota transplantation (FMT) is a revolutionary therapeutic strategy to restore the normal gut microbiota that has proven to be extremely effective in the treatment of *Clostridium Difficile* infection (140). It consists in one or more infusions of feces from a healthy donor into the gastrointestinal tract of the host *via* nasogastric or nasojejunal tube, upper or lower endoscopy or enema (141). According to a 2017 meta-analysis of 53 studies, FMT with multiple infusions administered through the lower gastrointestinal tract achieved a clinical remission rate of 36% in UC, 50.5% in CD and 21.5% in pouchitis and appeared safe with just transient gastrointestinal symptoms as adverse events (142). Data on the long-term effectiveness of FMT are controversial, but the response seems to decrease over 3 months; however, some patients exhibit long-term remission (143–146), pointing out that the therapeutic effect of FMT, particularly in patients with UC, is very promising, especially in those patients who received multiple transfusions. The effects of FMT in CD patients are currently being tested in several active trials. Some successful strategies to improve efficacy of FMT were identified and suggest that donor microbiota stability and species evenness, were highly relevant to efficacy and patient outcomes as well as specific microbial species could improve donor selection and build artificial microbiota for FMT (147). FMT seemed to be effective against some symptoms; however, further research needs to be conducted to improve the quality of data (148).

### Stem cell therapies

Stem cell therapy using hematopoietic stem cells (HSC) and mesenchymal stromal cells (MSC) is a promising strategy to improve IBD control, especially in refractory patients.

HSCs are defined as progenitor cells that are capable of unlimited self-renewal. HSC autologous transplantation (HSCT) has been suggested to reset the immune system, attenuating the abnormal inflammatory immune response in IBD patients. Based on this, several studies have been conducted on CD and UC patients, some of them using autologous HSCT on refractory CD patients. After one year of autologous HSCT, almost 40% of patients who received transplantation stopped immunosuppressive therapy compared with the control group (149). Despite this, HSCT has been discouraged as a treatment option for autoimmune conditions, in particular for patients with refractory CD, due to high mortality rates, complications and serious associated adverse events (150).

MSCs are a type of immature and undifferentiated stem cells and have a tissue regenerative role. They are pluripotent and able to transform into adipocytes, osteoblasts, myocytes, and chondroblasts. The immunosuppressive effect of MSCs on IBD is mainly due to the paracrine function that secretes different cytokines to inhibit inflammation. MSCs can also secrete exosomes to the intestinal mucosa to inhibit inflammation (151). The first animal experiment to study the role of MSCs in the treatment of a UC mouse model was conducted in 2006. The results demonstrated that bone marrow-derived MSCs played a role in repairing injured intestinal mucosa and in downregulating the immune function of T cells (152). In a phase III study, using adipose-derived MSCs, ASC transplantation (ASCT) for CD patients with complex perianal fistulas showed that 59.2% of ASCT-treated patients achieved remission compared to the placebo group and that adverse events were similar in both groups (153).

**Figure 2:**
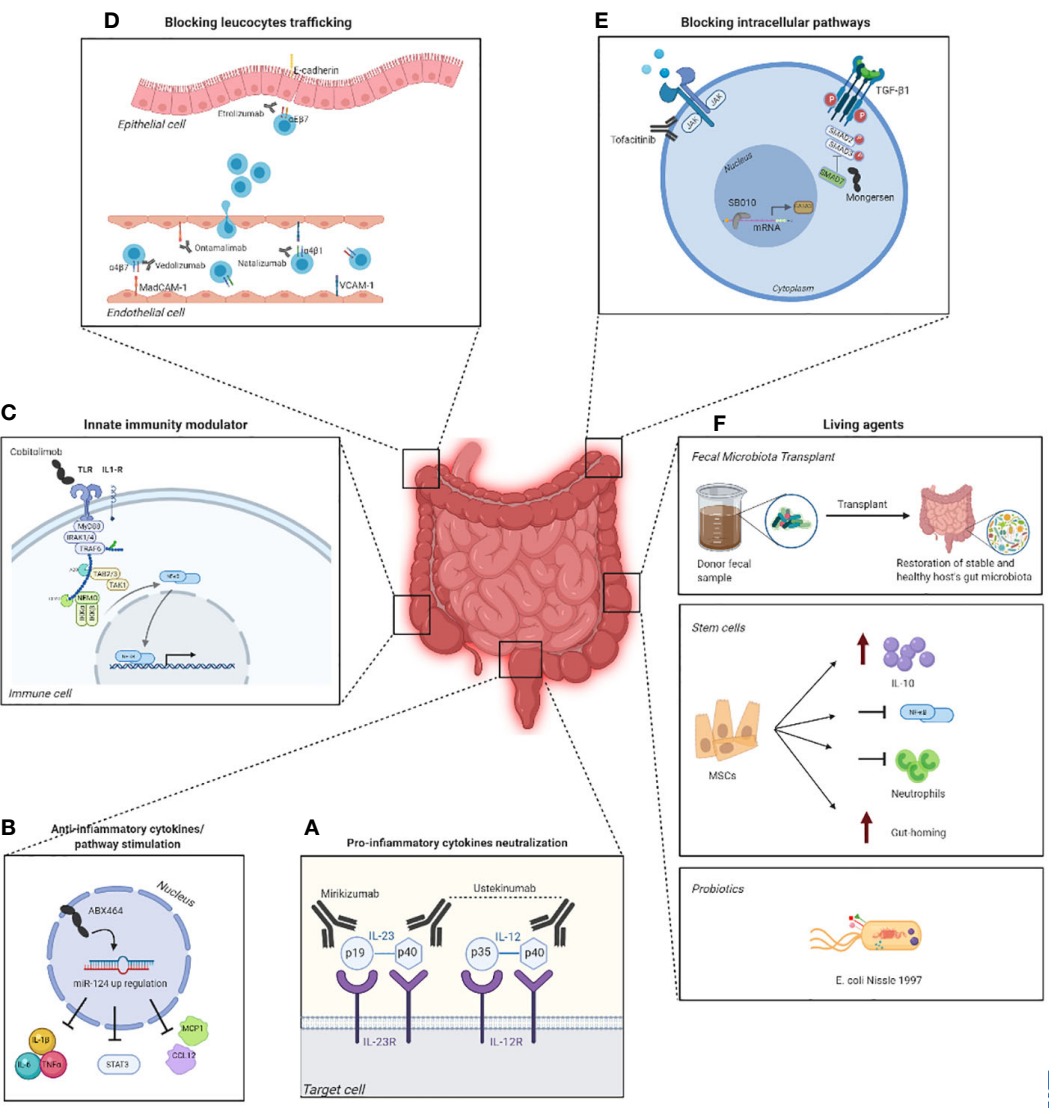
Emerging targeted therapies in IBD and their mechanism of action. Some therapies exploit monoclonal antibodies, such as mirikimumab and ustekinumab **(A)**, that inhibit the binding between pro-inflammatory cytokines and their respective receptor on the surface of the target cell, which in consequence blocks the development of the inflammation. However, there are molecules, such as ABX464, capable of modulating the production of anti-inflammatory targets to regulate the immune response **(B)**. Other therapies, on the other hand, aim to activate the innate immunity using drugs such as cobitolimod **(C)** that, by binding to the toll-like receptor 9 (TLR9) on the surface of the immune cell, leads to the suppression of Th17 cells and the induction IL-10^+^ macrophages and T_reg_ cells. Blocking the trafficking of the leucocytes in the epithelial cell is another valid therapeutic strategy and can be accomplished by a multitude of drugs like estrolizumab, vedolizumab, ontamalimab and natalizumab **(D)**, each of them designed to target a specific receptor. Another strategy is blocking intracellular pathways with drugs such as tofacitinib, that inhibits the JAK/STAT signaling pathway (panel **E**), and mongersen, which contrasts SMAD7 **(E)**. One last therapy revolves around the use of living agents as probiotics, fecal microbiota transplant (FMT) and stem cells that aim to increase the levels of IL-10 and the gut-homing and block neutrophils and the NFĸB pathway **(F)**. The figure was created with Biorender.com.

All these current and future therapies in IBD are shown in **Figure 2**.

## Complementary and alternative medicine

Complementary and alternative medicine (CAM) includes a very extensive and heterogeneous set of diagnostic-therapeutic practices that are not presently considered to be part of modern scientific medicine (24). Furthermore, the term ‘alternative medicine’ implies its use instead of, and the term ‘complementary medicine’ its use integrated with conventional medicine. CAMs have already been used extensively as coadjuvant in anti-cancer treatment; however, many publications show that CAMs are routinely used by patients affected by other categories of disease (154).

The percentage of IBD patients using CAM is high, ranging between 21% and 60% (23, 24). Most CAM therapies fall into one of the following main categories: herbal/botanical or dietary supplements; mind-body practices such as hypnosis, yoga/exercise, mindfulness and stress reduction. The most used CAM therapies are probiotics, herbs (e.g. curcumin, Chinese medicine, and cannabis) and fish oil (24). Other therapies include the use of traditional Chinese practices such as acupuncture and moxibustion.

Recent researches highlight that dietary therapy has a great implication for the treatment of IBD and suggest that the use of probiotics, dietary fibres and fat-soluble vitamins can substantially reduce the symptoms of IBD through their anti-inflammatory functions (155).

### Nutraceutical approaches

#### Non-starch polysaccharides

Non-starch polysaccharides (NPS), classified as dietary fibres and prebiotics, are obtained from various natural sources that have been studied extensively as therapeutics against inflammation and other immune-related problems. All of the major components of NPS, including cellulose, glucomannan, glucan, pectin, inulin, and oligosaccharides have exhibited anti-inflammatory and immunomodulatory functions (156). It has been hypothesized that the NPS components are fermented by probiotics in the healthy large intestine where they act by carrying out their anti-inflammatory function (157).

Konjac glucomannan is a plant-derived polysaccharide that has been used to treat gastrointestinal inflammatory disorders. For example, supplementation in IBD patients with konjac glucomannan hydrolysate for fourteen days resulted in improved bowel movement, fecal consistency and reduced abdominal pain, resulting in a better lifestyle (158).

#### Specific carbohydrate diets (SCD) and FODMAP

The specific carbohydrate diet (SCD) allows carbohydrate foods consisting of monosaccharides, and excludes foods containing disaccharides and most polysaccharides. Large-scale clinical trial data on SCD in IBD is lacking, but several studies have explored the role of SCD in IBD, particularly in paediatric patients (159).

In a clinical study, children with CD under this dietary plan for 12 and 52 weeks had remarkably reduced mucosal damage and improved clinical symptoms (160). Suskind and colleagues conducted a retrospective study of seven paediatric patients with CD and found that all patients experienced symptom remission within three months of initiating SCD, with either normalization or improvement in CRP, haemoglobin, albumin, and fecal calprotectin (161). Fermentable oligosaccharides, disaccharides, monosaccharides and polyols (FODMAP) is a term used to indicate sugars present in some highly fermentable foods, which can cause the onset of symptoms typical of an irritable bowel, e.g., abdominal swelling and pain, flatulence, alteration of the alvus or digestive difficulties. FODMAPs are short-chain carbohydrates that are not completely absorbed from the gastrointestinal tract, which can promote a state of excessive fermentation in the intestine that can compromise the quality of life in predisposed individuals. Evidence shows that a low FODMAP diet for 6 weeks helps to relieve symptoms of irritable bowel syndrome but there is currently no evidence to suggest that it works in IBD, although some patients find it helpful to control some of their symptoms (162).

#### Vitamins

It is also of interest to discuss the roles of fat-soluble vitamins such as A, D, E, and K, which are significantly decreased in patients with CD. Particularly, vitamin D deficiency in UC patients has been found to be associated with mucosal inflammation, and disease activity, which can increase the risk of clinical relapse of IBD (163, 164). The reasons for vitamin D deficiency are multifactorial but include lack of sun exposure due to immuno-suppressive treatments, dietary restrictions, and impaired nutrient absorption (165). Therefore, vitamin D supplements in IBD patients can result in positive outcomes. It has been demonstrated that vitamin D, by downregulating proinflammatory cytokines, IL-6, IL-21, TNF-α, and IFN-γ and by stabilizing the intestinal barrier, can contribute to the maintenance of the gastrointestinal barrier integrity and surveillance of the gut microbiota and inflammatory immune responses (166, 167).

#### Phytochemicals

Phytochemicals are substances naturally present in foods of plant origin and carry out important bioactive functions against oxidative and inflammatory disorders. Resveratrol is a polyphenol abundant in peanuts, berries and red grapes and exhibits biological functions that influence oxidative processes and inflammatory pathways. A randomized controlled study revealed that CD patients with resveratrol supplements had lower inflammation and reduced levels of TNF-α and NF-κB compared to the placebo group (168). The use of resveratrol efficaciously increased the activities of the antioxidant enzymes superoxide dismutase and glutathione peroxidase, protecting the cell from ROS damage. It can also reduce lipid peroxide and nitric oxide levels in colitis mice (169).

Acute colitis in an animal model was also partially inhibited by two other flavonoids, eupatilin and quercetin, which were orally administered 48 hours before colitis induction. The group of animals that received flavonoid extracts showed fewer mucosal lesions, nitric oxide and TNF-α production (170).

#### Prebiotics

Prebiotics are non-digestible organic substances, capable of selectively stimulating the growth and/or activity of one or a limited number of beneficial bacteria present in the colon. The best known and studied prebiotics are oligosaccharides and in particular inulin and fructo-oligosaccharides (FOS). Giving FOS to colitic mice resulted in increased lactic acid bacteria in the gut and decreased pro-inflammatory cytokines, such as IFN_-_γ, IL-17, and TNF-α (171). In addition, FOS treatment proved to have a protective effect on the epithelium, because both alkaline phosphatase (AP) enzyme activity and sensitivity to levamisole are decreased by FOS, which is consistent with a reduced AP isoform shift in enterocytes, a known feature of epithelial cells under inflammatory conditions.

#### Probiotics

Probiotics are live microorganisms (in most cases, bacteria) that are similarly beneficial to microorganisms present in the human gut and are used to restore gut bacterial flora. Probiotics ameliorate inflammation *via* several mechanisms, including alteration of the mucosal immune system, competitive exclusion of proinflammatory pathogens and production of antimicrobial factors such as bacteriocins and other metabolites (172). They also affect immune-modulatory pathways, such as downregulating the expression of Toll-like receptors and inflammatory cytokines as well as inhibiting the phosphoinositide 3-kinase/Akt pathway and the nuclear factor κ–light chain enhancer of activated B cells (NF-κB) pathway (173). They are also called “friendly bacteria” or “good bacteria”. Probiotics are available to consumers mainly in the form of dietary supplements and foods. Probiotics are among the most popular and most widely used CAM therapies and are available as single or multiple strains of bacteria or yeasts (24).

*Escherichia coli* Nissle (EcN) 1917 is a non-pathogenic Gram-negative strain used in many gastrointestinal disorders including diarrhoea, uncomplicated diverticular disease and IBD, in particular UC (174). Mechanisms of actions of this compound include immune-modulatory properties, reinforcement of intestinal barrier and inhibitory effect towards other pathogenic *E. coli (* 175).

*Lacticaseibacillus rhamnosus GG* proved to be effective and safe for maintaining remission and preventing relapse in patients with ulcerative colitis in a clinical trial from our group (176).

Other probiotics displayed interesting data in preclinical and in clinical settings, however, although we acknowledge several limitations to recent AGA guidelines on probiotics, more structured clinical trials are necessary to better position probiotics in therapeutical work up in IBD (177).

#### Herbaceous medications

Natural products, especially herbal medications and herbal extracts, have a relevant effect on the treatment of IBD (178) with numerous biological potencies, including anti-inflammatory activity (179).

Curcumin, or rather, Curcuma longa is a spice from the Zingiberaceae family. It has anti-inflammatory properties, which inhibit the two important mediators COX2 and TNF-α. Curcuma longa can reduce pro-inflammatory cytokines such as TNF-α, IFN-γ, IL-1β and IL-12, modulate signal transduction pathways such as p38MAPK and NF-kB, and reduce the expression of enzymes involved in the pathogenesis of inflammation (COX2) (180). Wang et al. found that different concentrations of curcumin could increase transepithelial electrical resistance by increasing the expression of occludins and maintaining the integrity of the tight junction (181). Curcumin, as a complementary therapy to 5-aminosalicylic acid (5-ASA), may be effective in inducing remission in mild to moderately active UC. In fact, a double-blinded, randomized pilot study documented the efficacy of curcumin in patients who were already on standard therapy and demonstrated a possible beneficial outcome in patients with distal UC and mild to moderate disease activity without significant side effects. Authors speculate that the use of curcumin can be a potential and safe therapy for the management of patients with UC (182).

Cannabis, a drug produced from the flowers and buds of the *Cannabis sativa* plant, has been therapeutically used for centuries (183). Cannabis contains dozens of terpene phenols known as cannabinoids many of which are pharmacologically active and induce their effects by binding to their specific CB1 and CB2 receptors. CB1 receptors are expressed in not only neurons of the central and peripheral nervous system but also in the GI gastrointestinal tract and are involved in various GI functions, such as: visceral pain, motility and sensitivity (184). CB2 receptors are mainly expressed in epithelial cells and cells of both innate and adaptive immunity, lymphocytes, macrophages, NK cells and neutrophils. Therefore, CB2 receptors are involved in the modulation of immune responses (180). Cannabinoids act on the endocannabinoid system, and have demonstrated analgesic and antinociceptive activity in several animal models and human (185). Cannabis has anti-inflammatory effects, and therefore may be useful in the treatment of several chronic inflammatory conditions including IBD (186). An unpublished meta-analysis of 100 patients from nonrandomized studies and randomized controlled trials until July 2018, considering the safety and efficacy of cannabis/cannabinoids in IBD was presented at the ECCO Congress 2019 (187). The authors found significant differences in disease activity scores in the treated group compared to the control group and an improvement in quality of life (QoL) and symptoms in cannabis-treated patients. However, data suggest a limited efficacy of cannabis in inducing remission of disease activity. In summary, cannabis may improve clinical symptoms but has not demonstrated any improvement in IBD activity. Aside from the lack of objective evidence that cannabis decreases inflammation in IBD, there are also legal and psychosocial risks involved with its use, particularly among younger patients (188). Side effects of cannabis use include confusion, ataxia, dizziness, nausea, and vomiting. Chronic use is associated with cognitive impairments and deficits in motivation, learning, and memory, as well as increased risk of motor vehicle crashes and decreased fertility (189). However, a recent study by Cocetta et al., showed that the non-psychotropic phytocannabinoid, cannabidiol isolated from *Cannabis sativa*, could represent the most promising candidate for clinical utilization due to its lack of psychoactive actions (190). The authors highlighted the role of cannabidiol as a potential modulator of markers of gut inflammation, being able to counteract the overproduction of reactive oxygen species and to prevent tight junction alterations allowing better maintenance of the intestinal epithelial barrier.

Fish oil is a dietary supplement derived from fish. It contains omega-3 fatty acids (n-3 PUFAs), which are one of the essential fatty acids in the human diet, along with omega-6 and omega-9. Oxidative stress occurs in several chronic inflammatory conditions, including IBD. Increasing the level of antioxidants could reduce tissue damage and the inflammatory process. Fish and fish proteins can have such antioxidant potential (191). Recently, a few studies completed in adults showed some common changes in the gut microbiota after omega-3 PUFAs supplementation. In particular, a decrease in *Faecalibacterium*, often associated with an increase in the *Bacteroidetes* has been shown. However, many more studies need to be conducted to demonstrate the effective relationship between omega-3 PUFAs consumption and gut microbiota changes (192).

#### Chinese herbal medicine

Herbal medicine (or phytotherapy) is a form of alternative medicine practiced for centuries, especially in China, where it is considered traditional medicine. For instance, *pomegranate peel decoction* can inhibit the growth of pathogenic intestinal bacteria (e.g. *C. difficile, Staphylococcus aureus* and *Pseudomonas ceruminous*), induce intestinal probiotics bacteria and regulate intestinal flora, improving its related disorders (193). The extract of pomegranate seeds is an oil that could inhibit proinflammatory cytokines’ expression (such as IL-6, IL-8, IL-23, IL-12, and TNF-α), preventing gastrointestinal inflammation (194). *Andrographis paniculata* (AP) is a traditional Chinese medicine that belongs to the *Acanthaceae* family and has anti-inflammatory properties on innate and adaptive immunity cells (e.g., dendritic cells, macrophages, T-cells, pro-inflammatory enzymes, cytokines and signal transduction pathways) (195). Andrographolide, an active ingredient isolated from the stem and leaves of AP, could have a down-regulating effect on the levels of TNF-α, IL-1β and IL-6 (196). AP extract (HMPL-004) and andrographolide sulfonate, an andrographolide derivative, could be employed respectively in the treatment of UC and experimental colitis (197). A recent study by Qin Zhu et al. investigated the andrographolide’s therapeutic effect in 2,4,6-Trinitrobenzene Sulfonic Acid Colitis (TNBS)-induced experimental colitis. Treatment with andrographolide showed a decrease in the percentages of Th17 cells in CD4+ cells and in pro-inflammatory cytokines’ levels (198).

*Indigo naturalis* (also referred to as Qing-Dai) is an herbal preparation extracted from leaves and stems of *Baphicacanthus cusia (Nees) Bremek*, which has been employed as a blue dye since ancient times (199). Its anti-inflammatory effect is supposed to be due to inhibition of TNF-α, IL-1 and IL-6, and NF-κB, as well as promotion of IL-22 production (200). In rat models with sodium dextran sulfate-induced colitis, *Indigo* reduced the expression of inflammatory cytokines while increasing the expression of proteins linked to the repair of the intestinal epithelium and colon mucosa (201). In a randomized, placebo-controlled trial, it was found that 8 weeks of *Indigo* was effective in inducing a clinical response in patients with UC. However, it should not yet be used because of the potential adverse effects, including pulmonary arterial hypertension (199).

*Pogostone* (PO) is one of the major chemical constituents of *Pogostemon cablin (Blanco) Benth.* In a study by Su et al., PO improved the inflammatory state, reducing effects on the infiltration of total Th cells into the inflammatory colon, especially pro-inflammatory Th1, and pro-inflammatory cytokine levels (202).

### Cognitive-physical approaches

#### Chinese practices (body-based interventions)

Acupuncture and moxibustion are therapies that have been used for over three millennia, representing the oldest practices of traditional Chinese medicine (203). Acupuncture consists in inserting thin needles into the skin at specific acupoints to attain various effects. Moxibustion consists in applying burning hot material on certain body areas, causing relief by the generated heat (203). A moxibustion’s variation is the herb-partitioned moxibustion (HPM), in which several herbs are added to burning cones to achieve the desired benefit (203). HPM turned out to promote the repair of damaged colonic tissue in ulcerative colitis rats and the secretion of mucin, which was correlated with an increased frequency of therapy (204). These clinical benefits were examined also in IBD patients in a meta-analysis, which assessed the clinical efficacy of acupuncture and/or moxibustion compared with sulphasalazine (SASP) for the treatment of UC (205). The overall efficacy of acupuncture alone, moxibustion alone or acupuncture and moxibustion combined was superior to the effectiveness of SASP.

#### Mind-body interventions

The role played by mood disorders and emotional distress on the microbiota is of growing interest. Studies in humans show that depression is associated with fecal microbiota alterations, with increased *Bacteroidetes*, *Proteobacteria* and *Actinobacteria*, and reduced *Firmicutes (* 206, 207). Furthermore, studies in animals proved that a fecal microbial transfer from depressed humans to non-depressed rats could trigger mood disorders in rats (208). The bi-directional relationship between mood and disease activity might evidence benefits IBD patients could obtain by integrating standard medical treatments with novel interventions focused on mental wellness (209). Since IBD and psychological stress are related, mind-body interventions (MBI) e.g., psychotherapy, relaxation, mindfulness, biofeedback, yoga and clinical hypnosis, as complement therapies, which target inflammatory disease activity, with safe, economic and non-pharmacological interventions might decrease stress response and increase the relaxation response of the autonomic nervous system (209).

Clinical hypnosis is a common MBI and refers to the specific ability to focus the patient’s attention narrowly deepening concentration, while simultaneously diminishing awareness of external stimuli. The mechanism of action of hypnotherapy is believed to be *via* the ‘brain–gut axis’, through the modulation of vagal visceral afferent signals. The strongest evidence of hypnotherapy’s effectiveness in IBD is its association with reduced IBD-related inflammation and improved QoL. Indeed, convincing studies have been performed suggesting that hypnotherapy can reduce inflammatory responses of the rectal mucosa (IL-6, IL-13, TNF-α, substance P and histamine) in patients with UC after just one hypnotherapy session (210).

Mindfulness is a type of meditation practiced to focus on the present moment, to relax in the “here” and “now”. It involves mind-body exercises where the patient tries to be fully engaged with a particular sensation, such as taste or smell, while focusing on breathing (211). In one trial, 60 patients with UC and CD were randomized to receive a mindfulness-based stress reduction intervention. The mindfulness intervention group for IBD patients reported significantly greater improvements in anxiety, QoL and mindfulness, with a significant reduction in depression compared to the control group (211).

Meditation is a practice of turning the patient’s attention inward calming the mind. There are many different forms of meditation, some including meditative movements such as Tai Chi or yoga.

Tai Chi is a martial art that has several beneficial effects on health, which is not only guaranteed by physical activity, but also by deep breathing and meditation exercises. Estaki et al. showed that cardiorespiratory fitness is associated with increased microbial diversity and butyrate production (212). Even though the association between Tai Chi and gut microbiota is still unknown, Tai Chi is supposed to modulate gut microbiota by improving cardiorespiratory fitness, which benefits the gut microbiota by reducing stress; it may also improve gut immune function and inflammation mediating the hypothalamic-pituitary-adrenal (HPA) axis and vagal modulation (213).

Yoga is an ancient Indian discipline, which affects the person physically, mentally and spiritually. In a RCT study, patients with UC in remission who performed 12 yoga sessions had an improvement in QoL, anxiety and abdominal pain, but no difference in their immune response markers was demonstrated (214).

**Figure 3:**
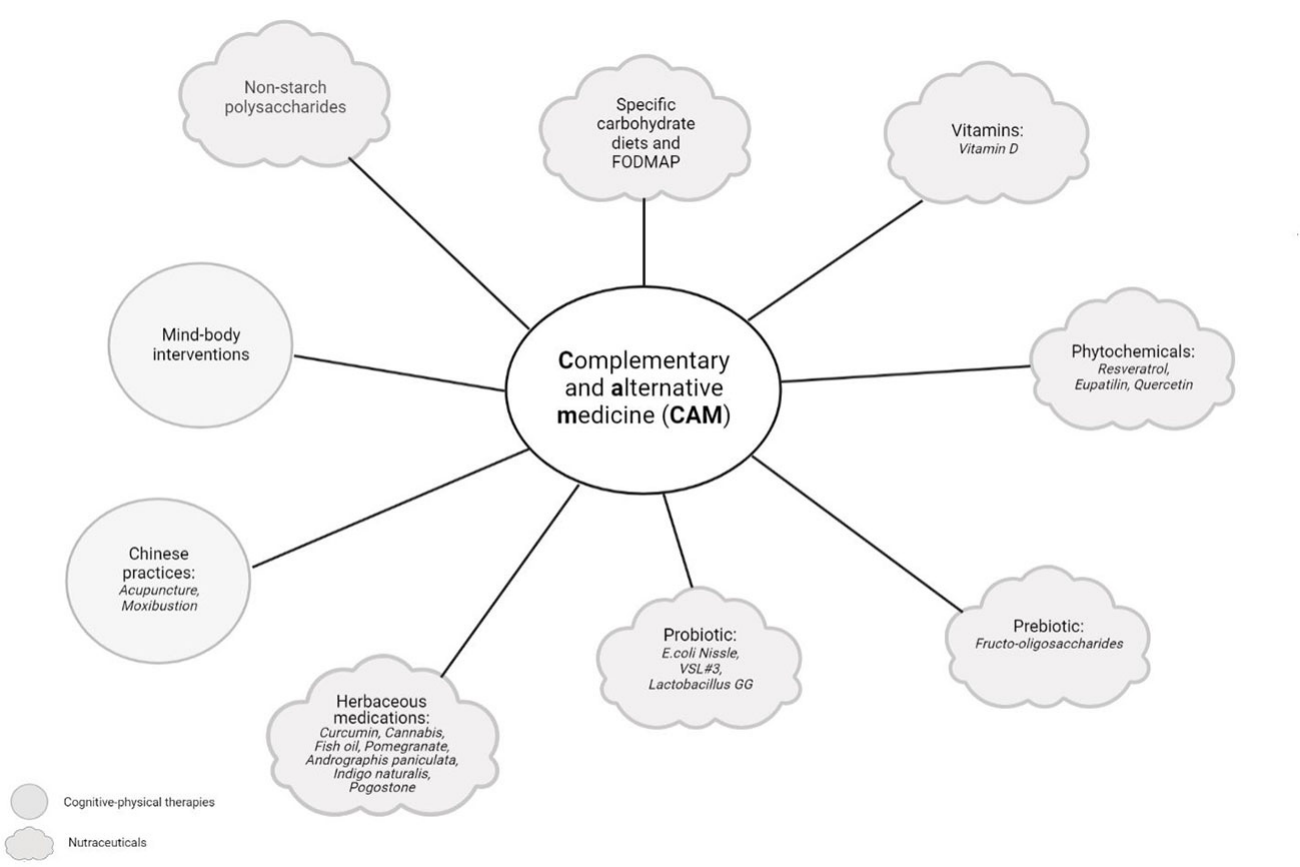
Complementary and alternative medicine (CAM) in IBD treatment. Complementary and alternative medicine (CAM) includes products or medical practices that encompass herbal and dietary supplements, probiotics, traditional Chinese medicines, and a variety of mind-body techniques. The figure was created with Biorender.com.

Physical activity in wider terms has been found to reduce some symptoms and complications of IBD, improving the QoL in patients (215). A study by Jones et al. demonstrated that patients with inactive CD who performed higher levels of exercise were less likely to develop active CD at 6 months. The study was replicated in UC patients, but the association was not statistically significant (216). In fact, strict exercise routines could be counter-productive in patients with active disease, since its interference with absorption could promote bleeding. In patients with chronic inflammation, exercise exerts an anti-inflammatory effect when IL-6 is released by the muscles, which inhibits the production of TNF and stimulates the release of IL-10. The release of myokine after physical activity has also been important in improving the anti-inflammatory effect (217). Physical activity has also counteracted bone mineral loss and targeted pain management and fatigue in patients with IBD. Other studies investigated the effects of exercise on inflammatory biomarkers like faecal calprotectin (FCP), C-reactive protein (CRP) and TNF-α, with merely one study showing a significant reduction in CRP (218). Despite the importance of TNF-α in IBD, only one study explored this parameter; however, no significant effect on the TNF-α basal level was observed within the treatment group that conducted mind-body therapy (219). **Figure 3** summarizes complementary and alternative medicine in IBD treatment.

## Discussion

IBD, with its unknown and multifactorial pathogenesis, has prevented the development of a unique therapeutic intervention that can be considered valid and effective for all affected patients. Furthermore, data from basic science suggest that IBD is an ensemble of several different diseases, which may require different therapeutic approaches depending on pathogenic features that are to date unknown.

For this reason, the 2020s “*IBDologist*” need to approach IBD more comprehensively, considering classic, innovative and complementary medicine.

In this comprehensive review, we have conferred a synthesis of current and investigational agents that are already enjoying or might play a very important role in the current and future treatment of IBD.

For many treatments, the results of RCTs or different varieties of clinical trials are the summit of several basic analysis studies that grow out of, contribute to, and improve our understanding of basic biological illness mechanisms. There is a crucial activity between basic analysis and clinical analysis. The basic analysis aims to advance data and understanding of the biological mechanisms of illness and treatment. A lot of clinical analysis builds on the results of the basic analysis to work out whether treatments supported new illnesses and whether treatment ideas will give measurable edges in specific patient populations. Findings from clinical analysis might support insights from basic analysis or reveal shocking results that cause new queries or hypotheses that are tested in laboratory studies. CAM come back from a range of sources and embrace seasoning therapies, organic process supplements, probiotics, and different physical or religious practices for the body and mind. though the majority contemplate these therapies as natural and safe, adverse effects will occur, and potential interactions with commonplace therapies ought to even be thought about. Patients usually address complementary and various medicines (CAM), which are medications or practices that are not a part of thought medication, to regulate their symptoms and manage their chronic ill health. Recent studies show patients with IBD use some variety of CAM to manage their illness (220–224). Given its increasing use in patients with IBD, physicians must treat these patients and remember current proof to raised counsel patients concerning the role of CAM in the management of IBD.

This review therefore highlights novel and emerging therapies for UC and CD and the most up-to-date results from clinical trials. We also report the data about unpublished paper related to undergoing clinical trials. However, there are a few limitations. The data from each study cannot be compared as all of the trials differ in design with varying inclusion and exclusion criteria, induction and maintenance periods, re-randomisation strategies following induction and outcomes. Limited data have been reported from negative and potential negative trials. No indications have been provided on where such treatments can be positioned into the treatments approach of active IBD patients.

Despite these limitations, our review provides an updated guide for gastroenterologists in classifying treatments, in order to facilitate decision making for IBD patients based on innovative, complementary and alternative therapy.

## Conclusion

The world of IBD is rapidly evolving while improving understanding of disease pathogenesis as well as increasing targets to different pathways.

In parallel to pharmacological therapies other complementary approaches (CAM) have also been developed, encountering the interest of a vast majority of patients, perhaps because of reported ‘natural’ (non -synthetic) origin and safety profile, whose use has also been assessed in clinical trials. We believe it is important that gastroenterologists take into consideration both pharmacological and CAM, in order to engage positively all patients. Furthermore, innovative therapeutic approaches coming from combination of CAM to other pharmacological approach are more than welcome, in order to maximise results believing in a perhaps synergic role of it.

Understanding impact and potential of combination therapies could drive the future of research in IBD to create a more personalised approach to the treatment of patients and improve outcomes.

## Author contributions

LMa, CC, and VP gave substantial contributions to the conception and design of the work. LMa, CC, and VP wrote the manuscript with the support of DP, CG, and LLo. PP, FdV, GP, MP, DN, LT, VA, and ES performed literature review. LP, LLa, LMi, MM, and AP have revised the work critically for important intellectual content. AA, AG, and FS made critical revisions related to the important intellectual content of the manuscript and gave the final approval of the article to be published.

## Acknowledgments

Thanks to Fondazione Roma for the continuous support to our scientific research.

## Conflict of interest

The authors declare that the research was conducted in the absence of any commercial or financial relationships that could be construed as a potential conflict of interest.

## Publisher’s note

All claims expressed in this article are solely those of the authors and do not necessarily represent those of their affiliated organizations, or those of the publisher, the editors and the reviewers. Any product that may be evaluated in this article, or claim that may be made by its manufacturer, is not guaranteed or endorsed by the publisher.

## Glossary

**Table:**

5-ASA	5-aminosalicylic acid
AP	Andrographis paniculata
ASCT	Adipose-derived mesenchymal stem cells transplantation
CAM	Complementary and alternative medicine
cAMP	Cyclic adenosine monophosphate
CB	Cannabinoid
CD	Crohn&rsquo;s disease
CHST15	Carbohydrate sulfotransferase 15
CRP	C-reactive protein
ECS	Endocannabinoid system
FCP	Faecal calprotectin
FMT	Fecal microbiota transplant
FODMAP	Fermentable oligosaccharides, disaccharides, monosaccharides and polyols
FOS	Fructo-oligosaccharides
GAG	Glycosaminoglycans
HPA	Hypothalamic-pituitary-adrenal
HPM	Herb-partitioned moxibustion
HRQOL	Health related quality of life
HSC	Haematopoietic stem cell
HSCT	Haematopoietic stem cell transplantation
IBD	Inflammatory bowel disease
ICAM-1	Intercellular cell adhesion molecule-1
IFN &alpha;	Interferon alpha
IL	Interleukin
JAK	Janus kinase
MadCAM-1	Mucosal vascular addressin cell adhesion molecule-1
MBI	Mind-body interventions
MSC	Mesenchymal stromal cells
NFkB	Nuclear factor kappa light chain enhancer of activated B cells
NKG2D	Natural Killer Group 2D
NPS	Non-starch polysaccharides
PAMP	Pathogen-associated molecular patterns
PDE4	Phosphodiesterase 4
PML	Progressive multifocal leukoencephalopathy
PO	Pogostone
PRO	Patient-reported outcome
PUFA	Polyunsaturated fatty acid
QoL	Quality of life
RCT	Randomized controlled trial
S1P	Sphingosine-1 phosphate
SASP	Sulphasalazine
SCD	Specific carbohydrate diets
STAT	Signal transducers and activators of transcription
TNBS	2,4,6-trinitrobenzene sulfonic acid colitis
TGF-&beta;	Transforming growth factor-beta
TLR	Toll like receptor
TNF	Tumor necrosis factor
UC	Ulcerative colitis
